# Alleviation of Plasma Homocysteine Level by Phytoestrogen *α*-Zearalanol Might Be Related to the Reduction of Cystathionine *β*-Synthase Nitration

**DOI:** 10.1155/2014/143192

**Published:** 2014-03-24

**Authors:** Hui Zhang, Qi Sun, Teng Liu, Lu Ma, Panpan Zhen, Ke Wang, Lingqiao Lu, Xin Liu, Xin Zhang, Dandan Song, Xiaoyun Zuo, Huirong Liu, Wen Wang

**Affiliations:** ^1^Department of Physiology and Pathophysiology, School of Basic Medical Sciences, Capital Medical University, No. 10 Xitoutiao, You An Men Wai, Beijing 100069, China; ^2^Beijing Key Laboratory of Metabolic Disorders Related Cardiovascular Diseases, No. 10 Xitoutiao, You An Men Wai, Beijing 100069, China; ^3^Department of Pathology, Beijing Luhe Hospital, Capital Medical University, 82 Xinhuanan Road, Tongzhou District, Beijing 101149, China; ^4^Beijing Stomatological Hospital, Capital Medical University, 4 Tiantanxili, Dongcheng District, Beijing 100050, China

## Abstract

Hyperhomocysteinemia is strongly associated with cardiovascular diseases. Previous studies have shown that phytoestrogen *α*-zearalanol can protect cardiovascular system from hyperhomocysteinemia and ameliorate the level of plasma total homocysteine; however, the underlying mechanisms remain to be clarified. The aim of this research is to investigate the possible molecular mechanisms involved in ameliorating the level of plasma homocysteine by *α*-zearalanol. By the successfully established diet-induced hyperhomocysteinemia rat models, we found that, after *α*-zearalanol treatment, the activity of cystathionine *β*-synthase, the key enzyme in homocysteine metabolism, was significantly elevated and level of nitrative stress in liver was significantly reduced. In correlation with this, results also showed a decreased nitration level of cystathionine *β*-synthase in liver. Together data implied that alleviation of plasma homocysteine level by phytoestrogen *α*-zearalanol might be related to the reduction of cystathionine *β*-synthase nitration.

## 1. Introduction

Hyperhomocysteinemia (HHcy) is considered as an independent risk factor for cardiovascular diseases [1]. Gene deficiency or poor activity of cystathionine *β*-synthase (CBS), the key enzyme involved in homocysteine metabolism, is a vital cause of hyperhomocysteinemia. Studies have demonstrated that estrogen could protect cardiovascular system from hyperhomocysteinemia-induced injuries [2]; nonetheless, the risk of developing breast and endometrial cancer in women taking estrogen has limited its clinical application [3–5]. Studies have documented that plant-derived phytoestrogens displayed cardiovascular protective effects similar to estrogen, meanwhile, without the potential negative effects that estrogen has [6]. Recently, *α*-zearalanol (*α*-ZAL), a plant-derived phytoestrogen, has been proposed as a potential substitute for estrogen, as it displayed a similar cardiovascular protective effect, without the negative side effects that estrogen showed [7]. *α*-Zearalanol is a reductive product of the fungus* Gibberella zeae* (*Fusarium roseum* Graminearum) metabolite zearalenone [8], and it is abundant in fruits and vegetables. Data from others and our group have revealed that *α*-zearalanol had certain cardiovascular protective effects but less side effects [9–13]. Our previous studies have demonstrated that *α*-zearalanol supplement not only attenuated homocysteine-induced vascular injuries but alsoalleviated plasma total homocysteine (tHcy) level in hyperhomocysteinemia rats [14]; however, the underlying mechanisms remain to be clarified. Celano et al. have reported that cystathionine *β*-synthase could be nitrated and consequently inactivated by peroxynitrite (ONOO^−^) in vitro [15]. Based on the previous findings, this study was designed to investigate the effect of *α*-zearalanol on cystathionine *β*-synthase activity and nitration. In this way we intend to explore the novel mechanism in cardiovascular protection of *α*-zearalanol and provide new ideas for the prevention and treatment of hyperhomocysteinemia.

## 2. Materials and Methods

### 2.1. Materials

3-Nitrotyrosine (NT) antibody ab110282 was purchased from Millipore (USA); cystathionine *β*-synthase (179–199) antibody sc-271886 and Protein A/G Plus-Agarose were purchased from Santa Cruz (USA); GAPDH antibody and HRP conjugate (GA1R) were purchased from THERMO SCIENTIFIC(USA); cystathionine *β*-synthase enzyme linked chemiluminescent kit was purchased from GENMED (USA); rat homocysteic acid (Hcy) ELISA kit was purchased from RapidBio (USA); *α*-zearalanol was purchased from Sigma-Aldrich (St. Louis, MO, USA).

### 2.2. Animals and Treatment Protocols

This investigation conformed to the Guide for the Care and Use of Laboratory Animals published by US National Institutes of Health (NIH publication number 85–23, revised 1996) and was performed with approval of the local institutional animal care and use Committee.

### 2.3. Group Distribution

Sixteen adult female Wistar rats (SPF grade) after ovariectomy were randomly divided into 2 groups: (1) OVX + Met (HHcy) and (2) OVX + Met + *α*-ZAL (*α*-ZAL + HHcy). HHcy rats were fed with 2.5% methionine diet for 16 weeks, and *α*-ZAL + HHcy rats were fed with 2.5% methionine diet plus *α*-ZAL for 16 weeks (2.5 mg/kg/day). The blood samples were collected from carotid arteries for tHcy detection. Livers were quickly removed for the NT and cystathionine *β*-synthase detection.

### 2.4. Measurement of Plasma tHcy by ELISA

The level of plasma tHcy was determined by commercial ELISA kit assay as in [14].

### 2.5. Measurement of Cystathionine *β*-Synthase Activity in Livers

After rats were anesthetized with 10% chloral hydrate, the livers were harvested. The cystathionine *β*-synthase activity in livers was determined by commercial enzyme linked chemiluminescent kit according to the instructions provided by the manufacturer.

### 2.6. Measurement of Cystathionine *β*-Synthase and NT Expression in Livers by Western Blot

Equal amounts of proteins from the liver were fractionated by 10% sodium dodecyl sulfate polyacrylamide gel electrophoresis (SDS-PAGE) and transferred onto a PVDF membrane. Nonspecific sites were blocked with 5% nonfat dry milk in trisbuffered saline with Tween 20 (TBS-T) for 1 hour at room temperature, following which the membrane was washed three times for 10 min each. The blots were then incubated over night at 4°C with appropriate primary antibody (mouse anti-cystathionine *β*-synthase 1 : 500, mouse anti-NT 1 : 1000, and mouse anti-GAPDH 1 : 8000) and then HRP-conjugated secondary antibody for 2 hours at room temperature. After washing by TBST three times for 15 minutes each, ECL Plus substrate (Thermo Scientific, Inc.) was applied to the blot, images were captured in a gel documentation system. Relative optical density of protein bands was analyzed using gel software image lab 3.0.

### 2.7. Measurement of NT Content in Livers by Immunohistochemistry

The livers were fixed in 4% paraformaldehyde for 4°C over night, dehydrated in ethanol, embedded in paraffin, and cross-sectioned (5 *μ*m). Parallel sections were subjected to standard immunohistochemical staining (mouse anti-NT 1 : 250 and goat anti-mouse IgG: 1 : 500).

### 2.8. Measurement of Nitrated Cystathionine *β*-Synthase in Livers 

For immunoprecipitation assay, 1 mg of protein extracts from each group was preincubated with 10 *μ*L protein G or A Sepharose for 2 hours and centrifuged at 2500 rpm for 5 min to withdraw nonspecific binding. 4 *μ*g of anti-cystathionine *β*-synthase or nonspecific mouse IgG antibody was added to the supernatant for 4 hours at 4°C, followed by incubation with 40 *μ*L protein G or A Sepharose over night at 4°C. The beads were washed three times with 500 *μ*L Wash Buffer I (1% Triton-X-100, 0.1% SDS, 50 mM HEPES, 150 mM NaCl, and PH 7.8), Wash Buffer II (1% Triton-X-100, 0.1% SDS, 50 mM HEPES, and PH 7.8), respectively, and bound proteins were eluted by boiling for 10 min with 5x SDS loading buffer, TTD, and analyzed by Western blot with anti-NT primary antibody, followed by anti-cystathionine *β*-synthase antibody.

### 2.9. Statistical Analysis

Data are expressed as mean ± SD or as percentages for categorical variables. Statistical analyses were performed using student *t*-test by SPSS. Results were considered significant if two sided *P* value was <0.05.

## 3. Results

### 3.1. *α*-Zearalanol Treatment Lowered Plasma tHcy Levels in HHcy Rats

**Figure 1:**
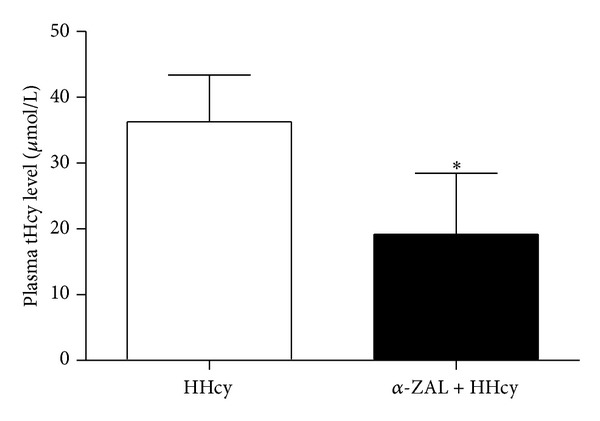
The total homocysteine level of rats' plasma (36.29 ± 7.09 µmol/L) was obviously above the normal level (15 µmol/L) after they were fed with 2.5% methionine diet for 16 weeks. *α*-Zearalanol treatment significantly lowered the total homocysteine level (19.20 ± 9.23 µmol/L). Data were expressed as mean ± SD; *n* = 8. Student *t*-test; *P < 0.05 versus HHcy. HHcy: hyperhomocysteinemia; tHcy: total homocysteine; *α*-ZAL: *α*-zearalanol.

Conventionally, hyperhomocysteinemia is defined as plasma tHcy above 15 *μ*mol/L. To compare the tHcy levels among each group of rats, we detected the level of tHcy by ELISA. Results showed that the tHcy levels of methionine diet-induced HHcy rats (36.29 ± 7.09 µmol/L) were obviously above the normal level (15 µmol/L). Meanwhile, *α*-zearalanol treatment significantly lowered the tHcy levels (19.20 ± 9.23 µmol/L) when compared with HHcy (*P* < 0.05, Figure 1).

### 3.2. *α*-Zearalanol Treatment Increased Cystathionine *β*-Synthase Activity but Not Expression in the Livers of HHcy Rats

**Figure 2:**
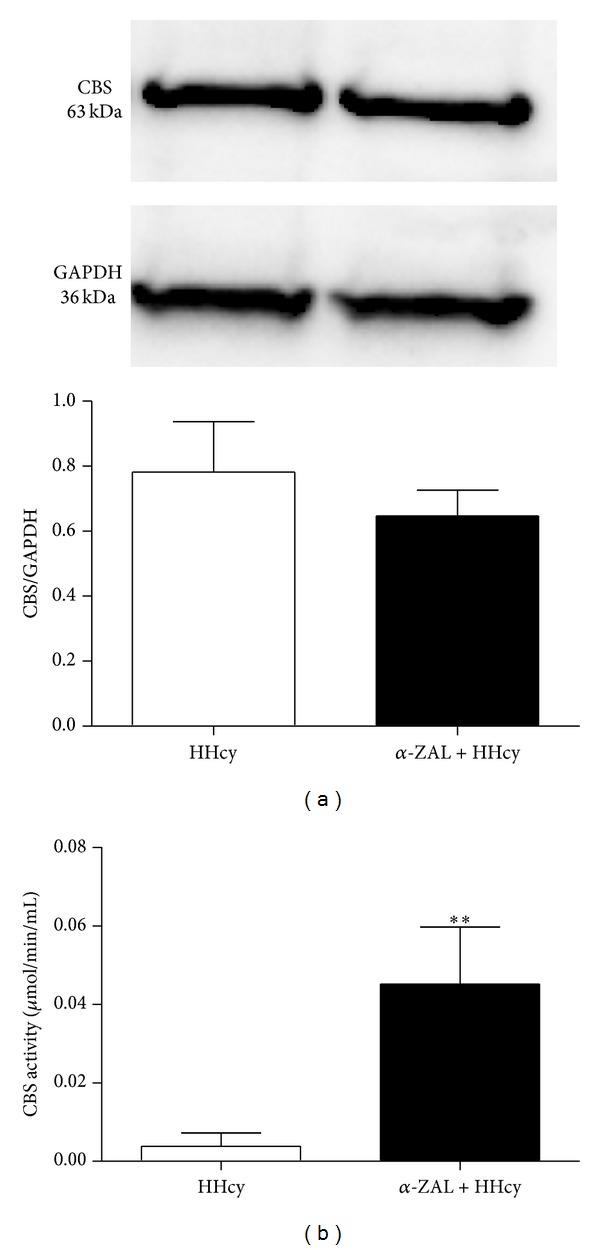
*α*-Zearalanol treatment increased cystathionine *β*-synthase activity in the liver of HHcy rats. (a) The cystathionine *β*-synthase expression was detected by Western blot and normalized by GAPDH expression. (b) The cystathionine *β*-synthase activity was detected by enzyme linked chemiluminescent kit. Data were expressed as mean ± SD; *n* = 3. Student *t*-test; **P < 0.01  versus HHcy. CBS: cystathionine *β*-synthase; HHcy: hyperhomocysteinemia; *α*-ZAL: *α*-zearalanol.

To explore the effects of *α*-zearalanol oncystathionine *β*-synthase in HHcy rats, we measured the cystathionine *β*-synthase expression and bioactivity of each group. Interestingly, the cystathionine *β*-synthase expression of each group displayed no significant difference (*P* = 0.44) (Figure 2(a)), whereas *α*-zearalanol treatment increased the activity of cystathionine *β*-synthase (*P* < 0.01 versus HHcy, Figure 2(b)).

### 3.3. *α*-Zearalanol Treatment Decreased 3-Nitrotyrosine Content in the Livers of HHcy Rats

**Figure 3:**
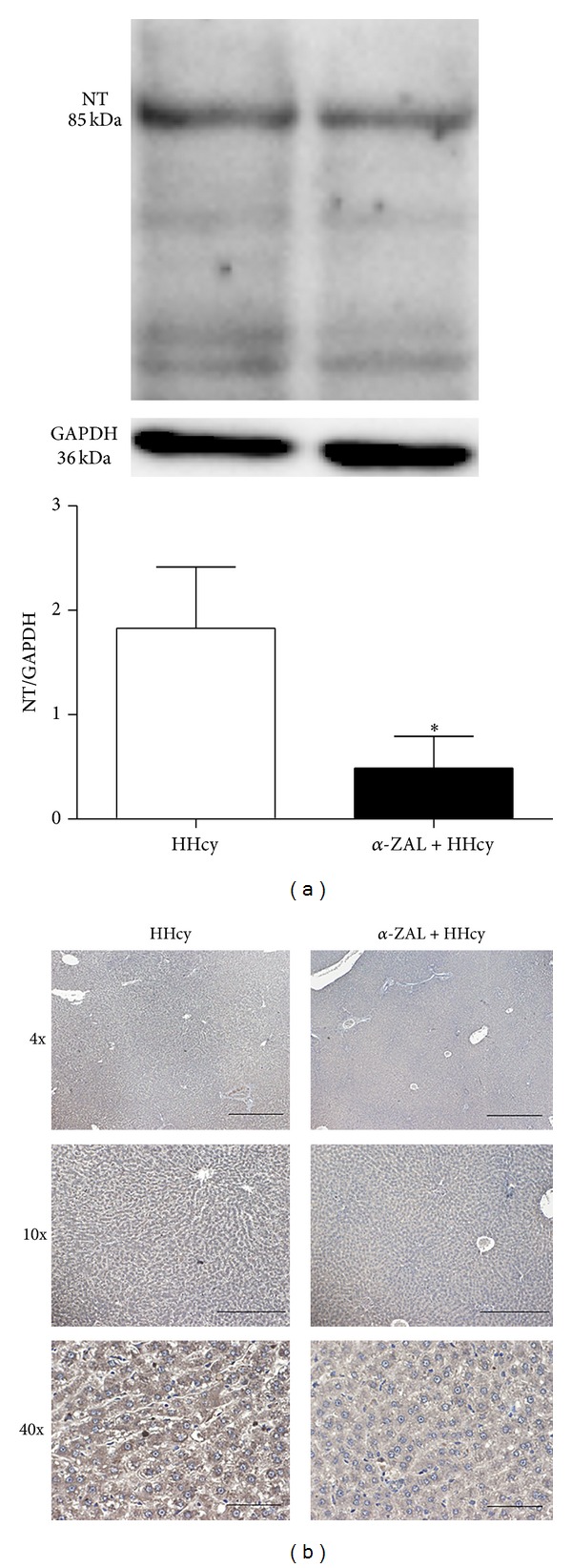
*α*-Zearalanol treatment decreased 3-nitrotyrosine content in the livers of HHcy rats. The 3-nitrotyrosine content was detected by Western blot (normalized by GAPDH expression) (a) and immunohistochemistry (b). Data were expressed as mean ± SD; *n* = 3. Student *t*-test; *P < 0.05 versus HHcy. HHcy: hyperhomocysteinemia; NT: 3-nitrotyrosine; *α*-ZAL: *α*-zearalanol.

3-Nitrotyrosine (3-NT) would be generated when protein is modified by peroxynitrite; thus it is commonly used to reflect the level of peroxynitrite in vivo. It was previously reported that cystathionine *β*-synthase could be nitrated and consequently inactivated by peroxynitrite (ONOO^−^) in vitro. To explore whether *α*-zearalanol could influence the level of peroxynitrite or not, we further observe the effect of *α*-zearalanol treatment on NT content in the livers of HHcy rats. Result showed that with *α*-zearalanol treatment, there was a significant attenuation of NT content in HHcy rats (*P* < 0.05 versus HHcy, Figure 3), implying that the high level of nitrative stress could be reduced by *α*-zearalanol.

### 3.4. *α*-Zearalanol Treatment Attenuated Cystathionine *β*-Synthase Nitration in the Livers of HHcy Rats

**Figure 4:**
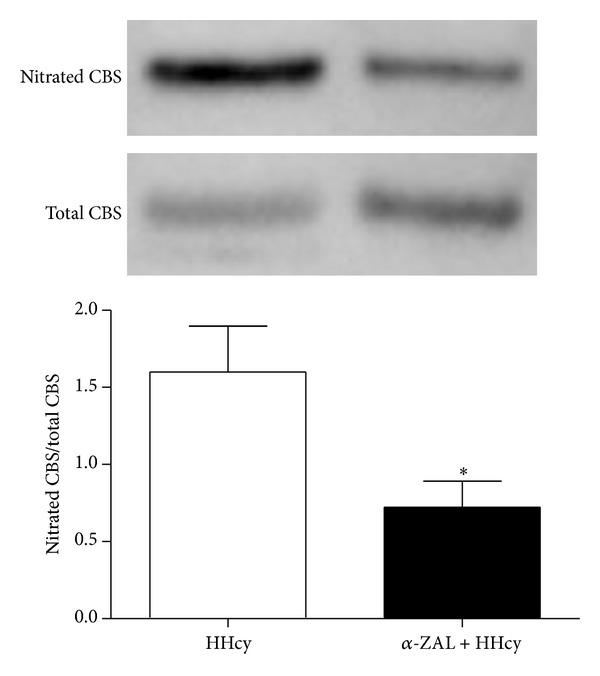
*α*-Zearalanol treatment attenuated cystathionine *β*-synthase nitration in the livers of HHcy rats. Nitrated cystathionine *β*-synthase in liver tissue was detected by immunoprecipitation along with Western blot. Data were expressed as mean ± SD; *n* = 3. Student *t*-test; *P < 0.05 versus HHcy. CBS: cystathionine *β*-synthase; HHcy: hyperhomocysteinemia; *α*-ZAL: *α*-zearalanol.

Based on the above findings, furthermore, we explored the effect of *α*-zearalanol on cystathionine *β*-synthase nitration by immunoprecipitation along with Western blot. As expected, our results showed that *α*-zearalanol treatment significantly attenuated cystathionine *β*-synthase nitration in the livers of HHcy rats (*P* < 0.01 versus HHcy, Figure 4), further implying that *α*-zearalanol might protect cystathionine *β*-synthase bioactivity by inhibiting cystathionine *β*-synthase protein nitration in HHcy rats and consequently preventing tHcy elevation.

## 4. Discussion

Homocysteine is a sulfur-containing amino acid derived from the dietary amino acid methionine [16]. Methionine can be metabolized into homocysteine in vivo, and high methionine diet can cause overaccumulation of plasma total homocysteine (tHcy). Here we fed rats with 2.5% methionine diet for 16 weeks to duplicate the hyperhomocysteinemia models. To exclude the influence of endogenous estrogen, we intentionally ovariectomized all the rats before duplicating the hyperhomocysteinemia models. And in agreement with our previous study [14], this time our result again showed that *α*-zearalanol treatment could significantly attenuate tHcy in HHcy rats. Why did this happen? What would be the underlying mechanisms?

In mammals, homocysteine is formed in the methionine metabolism cycle and catalyzed by cystathionine *β*-synthase in the transsulfuration pathway. Since transsulfuration pathway metabolizes homocysteine via an irreversible pathway which forbids reconstruction, its key enzyme cystathionine *β*-synthase is more essential than other enzymes in terminal removement of homocysteine. Recent studies have demonstrated that deficiencies in cystathionine *β*-synthase expression or activity contribute to hyperhomocysteinemia, and vitamin B_6_, the coenzyme of cystathionine *β*-synthase, treatment alone cannot decrease homocysteine [17, 18]. Thus, improving the expression or activity of cystathionine *β*-synthase might be the new target in the future. In this study, we found out that although cystathionine *β*-synthase expression exhibited no significant difference among each group, it was noteworthy that in HHcy rats *α*-zearalanol significantly increased cystathionine *β*-synthase activity, indicating a protective effect of *α*-zearalanol on cystathionine *β*-synthase activity. How can *α*-zearalanol protect cystathionine *β*-synthase activity?

Cystathionine *β*-synthase contains three functional domains and the middle domain accounts for the pyridoxal phosphate-catalyzed reaction; the N-terminal domain is responsible for redox conditions; the C-terminal domain contains S-adenosylmethionine negative regulatory region that uniquely regulates allosteric activation of the enzyme, which is essential in the metabolism of homocysteine. Thus, some translational modification might result in cystathionine *β*-synthase inactivation and homocysteine metabolic imbalance. Studies have claimed that hyperhomocysteinemia could lead to disrupted nitric oxide (NO) signaling that contributes to the loss of NO bioavailability [19], and the accumulation of superoxide anion which accompanies free NO [20]. As an outcome, the interaction of superoxide anion with NO generates peroxynitrite (ONOO^−^), and the excessive generation of ONOO^−^ triggers nitrative stress. Nitrative stress may induce the nitrative modification of proteins, which lead to different injuries like cardiovascular dysfunction, inflammatory lung diseases, and Parkinson's disease. ONOO^−^ is one of the most vital reactive nitrogen species (RNSs) in nitrative stress; it can barely be detected due to its unstable feature. A characteristic reaction of ONOO^−^ is the nitration of protein-bound tyrosine residues to produce 3-nitrotyrosine, and the later has been used extensively as a footprint for ONOO^−^in vivo [21, 22]. In our data set, the 3-nitrotyrosine content is much lower in HHcy rats with *α*-zearalanol treatment, implying that the high level of nitrative stress can be reduced by *α*-zearalanol in HHcy rats. In correspondence with this, result from immunoprecipitation along with Western blot assay displayed the fact that *α*-zearalanol treatment significantly alleviated the level of cystathionine *β*-synthase protein nitration, further implying that *α*-zearalanol might protect cystathionine *β*-synthase bioactivity by inhibiting cystathionine *β*-synthase protein nitration in HHcy rats and consequently preventing elevation of tHcy.

Taken together, in this study, firstly we indicated that *α*-zearalanol alleviated plasma total homocysteine via protecting cystathionine *β*-synthase bioactivity in HHcy rats. Furthermore, we found that the improvement of cystathionine *β*-synthase bioactivity by *α*-zearalanol might be related to the reduction of cystathionine *β*-synthase nitration. Although it is possible that nitration of other enzymes involved in homocysteine metabolism may also take roles in hyperhomocysteinemia development, and the detailed molecular mechanisms responsible for *α*-zearalanol still remain to be explored, this new insight gained from the current study may shed a novel light on better mechanistic understandings of *α*-zearalanol and provide new ideas for the prevention and treatment of hyperhomocysteinemia.
